# A Novel Modulation Classification Approach Using Gabor Filter Network

**DOI:** 10.1155/2014/643671

**Published:** 2014-07-14

**Authors:** Sajjad Ahmed Ghauri, Ijaz Mansoor Qureshi, Tanveer Ahmed Cheema, Aqdas Naveed Malik

**Affiliations:** ^1^ISRA University, Islamabad 44000, Pakistan; ^2^School of Engineering & Applied Sciences (SEAS), ISRA University, Islamabad Campus, I/10 Markaz, Islamabad 44000, Pakistan; ^3^International Islamic University, Islamabad 44000, Pakistan; ^4^AIR University, Islamabad 44000, Pakistan; ^5^Institute of Signals, Systems and Soft Computing (ISSS), Islamabad, Pakistan

## Abstract

A Gabor filter network based approach is used for feature extraction and classification of digital modulated signals by adaptively tuning the parameters of Gabor filter network. Modulation classification of digitally modulated signals is done under the influence of additive white Gaussian noise (AWGN). The modulations considered for the classification purpose are PSK 2 to 64, FSK 2 to 64, and QAM 4 to 64. The Gabor filter network uses the network structure of two layers; the first layer which is input layer constitutes the adaptive feature extraction part and the second layer constitutes the signal classification part. The Gabor atom parameters are tuned using Delta rule and updating of weights of Gabor filter using least mean square (LMS) algorithm. The simulation results show that proposed novel modulation classification algorithm has high classification accuracy at low signal to noise ratio (SNR) on AWGN channel.

## 1. Introduction

Digital modulation is an important factor in communication system. Identification of received signal modulation in the presence of channel noise in noncooperative communication is a complex issue. Before demodulation of received signal, modulation classification is done. Modulation classification is a technique that allows receiver to become cognizant of current status of transmitted data and channel. Applications of modulation classification (MC) are in commercial sector (interference identification, spectrum management), military domain, and software defined radio (SDR). If receivers classify the modulation scheme successfully, the SDR can be used as a receiver for modification of demodulation part. The MC has applications also in cognitive radios (CR).

After the detection of received signal and before the demodulation of received signal, modulation classification process is done. Modulation classification process involves two steps; the first is feature extraction of the transmitted signal and the second is classification of the signal based upon feature extraction. In the literature, various methods have been proposed for classification/identification of different modulation formats [1]. Basically, automatic modulation classification process is distributed in two approaches [2]: (1) log likelihood function based decision theoretic approach; (2) extraction of features based pattern recognition approach.

Log likelihood function based decision theoretic approach has been proposed in [3–5]. This approach is basically a function of symbols which are to be transmitted, as well as channel parameter. The log likelihood function is calculated under each modulation format (hypothesis). The modulation format which maximizes the likelihood function is the decision. The log likelihood function based decision algorithms are optimal because they minimize the probability of error in classification of signal, but they are computationally complex. The log likelihood function based decision theoretic approach requires a priori knowledge about the signal. The likelihood functions become tussle and hard to implement because of channel conditions. Extraction of features based pattern recognition approach is also known as feature based (FB) approach. The decision is made on the observation of the extracted feature set. FB approach is suboptimal method with reasonable computational complexity as compared to log likelihood based algorithms and also easy to implement [6, 7].

In the literature, the feature which was extracted from the transmitted signal is of many types such as higher order moments (HOM), higher order cummulants (HOC) up to 8th order (HOM) [1], spectral features (*σ*_ap_, *σ*_dp_, *σ*_aa_, *σ*_af_, *σ*_fn_, and *γ*_max⁡_) [8], and cyclic features (spectral coherence function, cyclic domain profile) [9]. In [1, 10], authors present the automatic modulation classification algorithm which uses HOC for estimation of channel and pattern recognition; no a priori information is required. In [11], HOC up to 4th order are used for classification of modulations over an AWGN channel. The features utilized in [9, 12, 13] are spectral features (*σ*_ap_, *σ*_dp_, *σ*_aa_, *σ*_af_, *σ*_fn_, and *γ*_max⁡_) and cyclic features (spectral coherence function, cyclic domain profile). A summary of proposed algorithms based on time-frequency analysis, wavelet transform, higher order statistics (cummulants and moments), cyclo-stationarity properties and on spectral properties for modulation classification are in [14].

The modulation identification of BPSK, QPSK, 16-PSK, 2-16QAM, GMSK, and MSK modulations schemes under the effect of noisy channel is considered in [15], using wavelet transform approach and statistical moments as features. The proposed algorithm performs 100% identification at SNR of 10 dB. A hierarchical cyclostationary based algorithm is proposed to identify the wide range of unknown modulated signals presented in [16]. The authors also assume no a priori information such as carrier frequency and carrier phase. The modulation formats to be classified are AM, BPSK, OFDM, CDMA, 4–8 ASK, 2–16 PSK, 16, and 64 QAM modulation formats. The performance of proposed algorithm is investigated on fading channels. The proposed algorithm in [17] uses higher order moments of continuous wavelet transform (CWT) as a feature set. The classifier used is multilayer feed forward neural network using resilient back propagation algorithm. The modulation formats considered are M-ary shift keying without any priori information. The performance of algorithm is evaluated on AWGN channel as well as fading channels. In [18], FSK and AM signals are jointly detected and classified using first order cyclostationarity. The algorithm only requires approximate information about signal band width and carrier frequency and there is no need for time recovery in the proposed algorithm. In [19], features are extracted based upon autocorrelation and cyclic autocorrelation for cyclic prefix guard time interval OFDM signals to estimate the useful time interval, the cyclic prefix duration, and the number of subcarriers in frequency selective channels. Authors use time frequency analysis to extract features in [20]. These extracted features are used for classification purpose. The proposed algorithm has three main tools; a TF tool which computes the TF transform, extraction of features which gives main characteristics of signal, and classifier part which discriminates with the help of features. The modulation classification for the wireless sensor networks is carried out in [21] using multisensor fusion based method. The classification performance is investigated on AWGN channel and fading channel. The a priori information about the signal such as timing synchronization, phase jitter, phase offset, and frequency offset is considered for evaluation of correct classification. The first signal classification is done using 2nd, 4th, and 6th order cummulants and then kernel thought is used to map the feature to higher dimensional space and optimum hyper plane is constructed using SVM to classify the signals in [22]. The classifier based on back propagation neural network (BPNN) and trained by improved particle swarm optimization (PSO) which is used to optimal weights and threshold for BPNN is proposed in [23]. The recognized modulation formats are 2ASK, 4ASK, 2FSK, 4FSK, BPSK, and QPSK. The classifier designed for space time block codes (STBC) system using multidimensional independent component analysis (ICA) is proposed in [24]. The classifier is also based on maximum likelihood on the condition of virtual channel matrix. The features extracted for modulation identification are received signal power distribution in [25]. The classifier identifies only six modulation formats. Higher order cummulants up to 4th and 6th order are used to classify the modulation formats such as BPSK, QPSK, and higher order QAM's [26]. Cyclostationary features are also used to classify the BPSK and non-BPSK, at last maximum likelihood detection algorithm is performed to further classification of QPSK and 16-QAM. The modulation classification is done without having any information about noise power, energy of the transmitted signal, and channel coefficients [27]. The modulation classification is carried out by minimizing the distance of log likelihood and expected log likelihood of the received data. Modulation classification is considered on frequency selective fading channel using Gibbs sampling method based on latent Dirichlet Bayesian network in [28]. Blind modulation classification problem is considered under the effect on frequency selective fading channels in [29]. The features used for classification are correlation function of the received signal. The modulation recognition focuses on pattern recognition method proposed in [30]; the main purpose is to demonstrate the possibility of recognizing digital modulation formats at lower SNRs. The method of modulation classification involves computation of the empirical characteristics function (ECF) from the received signal samples and employing constrained least squares (CLS) filtering in frequency domain [31].

For signal classification and representations, adaptive time frequency analysis including wavelet based filter bank [32] and Gabor based filter banks were used in [33, 34]. Recently, in [35], authors proposed a Gabor atom based upon neural network (GNN) for feature extraction and signal classification. Efficient feature extraction and higher recognition rate have been achieved using GNN. However, GNN consists of two layers; the first layer is feature extraction and the second layer is of signal classification. Using degree of non-stationarity, the Gabor filter is proposed in [36], for efficient classification.

In this paper, the authors have proposed joint approach for feature extraction and classification for multisignal vector. Gabor filter based approach is used to classify the digital modulated signal in the presence of AWGN channel. The Gabor filter parameters are adjusted adaptively using the Delta rule. The weights of the adaptive filter are adjusted using least mean square (LMS) algorithm. The digital modulations considered in this paper are PSK2, PSK4, PSK8, PSK16, PSK32, PSK64, FSK2, FSK4, FSK8, FSK16, FSK32, FSK64, QAM2, QAM4, QAM8, QAM 16, QAM 32, and QAM 64. The proposed algorithm gives high classification accuracy at lower SNRs. The mean square error (MSE) for training of Gabor filter network versus number of iterations as well as versus SNR for considered modulations is also shown. The simulation results for testing of proposed algorithm show high classification accuracy.

The rest of the paper is organized as follows.  Section 2 represents the system model.  Section 3 represents the Gabor filter for classification and feature extraction. In Section 4, Gabor filter training and testing algorithm is presented.  Section 5 discusses the performance of proposed classifier in the presence of AWGN, while the whole paper is concluded in Section 5.

## 2. System Model

The generalized expression for signal received is given by

(1) $$\begin{array}{c} r\left(n\right)=x\left(n\right)+g\left(n\right), \end{array}$$

where *r*(*n*) is complex baseband envelop of received signal, *g*(*n*) is the additive white Gaussian noise with zero mean and a variance of *σ*_*g*_^2^, and *x*(*n*) is given by

(2) $$\begin{array}{c} x\left(n\right)=\alpha e^{i(w_{o}nT+\theta_{n})}\overset{j=\infty }{\underset{j=-\infty }{\sum }}x\left(l\right)h\left(n\tau -j\tau +\epsilon_{T}\tau \right), \end{array}$$

where *x*(*l*) = input symbol sequence which is drawn from set of *M* constellations of known symbols and it is not necessary that symbols are equiprobable, *α* = amplitude of signal, *w*_*o*_ = angular frequency offset constant, *τ* = symbol spacing, *θ*_*n*_ = the phase jitter which varies from symbol to symbol, *h*(⋯) = channel effects, and *ϵ*_*T*_ = the timing jitter.

**Figure 1:**
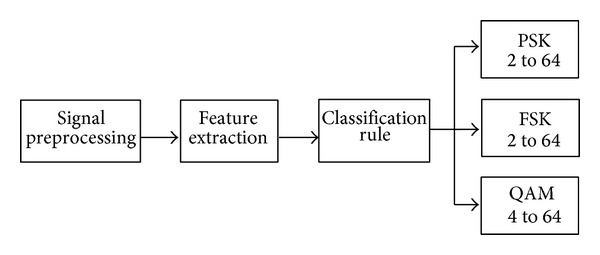
System model for modulation classification.

The system model for classification of modulation signals is shown in Figure 1. First feature extracted from the received signal which is corrupted by additive white Gaussian noise, after extraction of these features the classification is based upon the feature extracted. The received signal may be PSK, FSK, or QAM modulated.

## 3. Gabor Filter for Classification and Feature Extraction

Gabor atom is efficient tool for feature extraction. The Gabor atom in simple form can be written as

(3) $$\begin{array}{c} g_{c,\sigma ,f}\left(t\right)=\frac{1}{\sqrt{\sigma }}g\left(\frac{t-c}{\sigma }\right)e^{jft}, \end{array}$$

where *g*(*t*) = 2^1/4^ *e* ^−*πt*^2^^and *c*, *σ*, and *f* are shift parameter, scale parameter, and modulation parameter, respectively.

**Figure 2:**
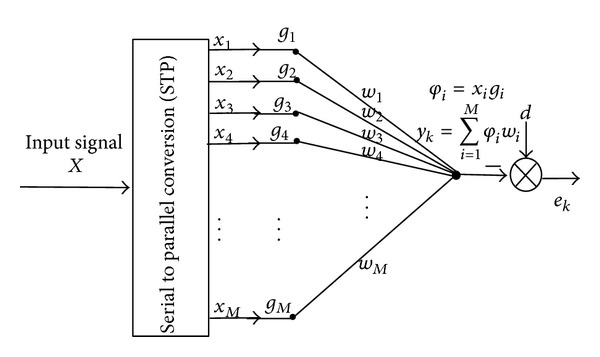
Gabor filter network with input layer which is feature extraction part; weights and output layer are linear classification part.

In Figure 2, Gabor filter network is shown which has two-layer filter. The input to Gabor filter network is first serial to parallel converted {*x*_*i*_, *i* = 1,2, 3,…, *M*} and outputs are {*y*_*k*_, *k* = 1,2, 3,…, *N*}. Let {*g*_*i*_, *i* = 1,2, 3,…, *M*} be the *i*th class Gabor atom and be defined as

(4) $$\begin{array}{c} g_{i}\left(t\right)=\frac{1}{\sqrt{\sigma_{i}}}g\left(\frac{t-c_{i}}{\sigma_{i}}\right)e^{jf_{i}t}. \end{array}$$

The Gabor atom parameters (*c*, *σ*, and *f*) are required to be adjusted until some cost function is minimized.

The input layer has *M* nodes *φ*_1_, *φ*_2_, *φ*_3_,…, *φ*_*M*_ also called Gabor nodes. The output of the *i*th Gabor atom node is *φ*_*i*_ corresponding to input signal *x*_*i*_. Thus, output of Gabor atom is defined as

(5) $$\begin{array}{c} \varphi_{i}=\left|\langle {g_{i},x}_{i}\rangle \right|,\\ \varphi_{i}=\left|\int \frac{1}{\sqrt{\sigma_{i}}}g^{*}\left(\frac{t-c_{i}}{\sigma_{i}}\right)e^{-jf_{i}t}x_{i}\left(t\right)dt\right|. \end{array}$$

The output layer consists of *N* nodes {*y*_*k*_, *k* = 1,2, 3,…, *M*} and for convenience *N* is usually set to 1. The output of the Gabor atom node *φ*_*i*_ in the input layer is weighted by *w*_*i*_; that is,

(6) $$\begin{array}{c} y_{kn}=\overset{M}{\underset{i=1}{\sum }}\varphi_{in}w_{in},\;n=1,2,3,\ldots ,N. \end{array}$$

Gabor filter network consists of two layers: the input layer is feature extraction and the second layer has Gabor filter weights which constitutes the linear classification part.

Feature extraction using Gabor filter network is that both Gabor atom parameters and Gabor filter weights are adjusted to minimize the sum of squared error. The difference between the desired outputs *d*_*k*_ and actual output of Gabor filter *y*_*k*_ is defined as

(7) $$\begin{array}{c} e_{k}{=d}_{k}-y_{k}. \end{array}$$

In [35], the Gabor atom parameters and neural network weights are adjusted simultaneously in training phase. Such a joint updating of Gabor atom parameters and neural networks weights show some deficiencies. These deficiencies will cope when Gabor atom parameters and neural networks weights are adjusted separately [36].

In training phase of modulation classification, the two adaptive algorithms are performed by Gabor filter network: (1) the updating of Gabor atom parameters (*c*, *σ*, and *f*); (2) for given set of Gabor atom parameters, algorithm updates the weight of Gabor filter.

**Figure 3:**
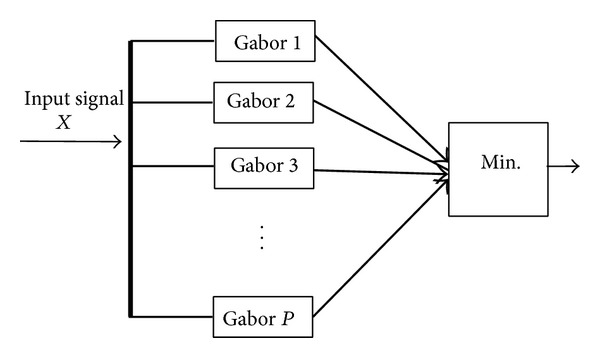
Testing scheme for modulation classification.

In testing phase, shown in Figure 3, the modulated signal may be PSK 2 to 64, FSK 2 to 64, and QAM 2 to 64. The modulated signal is passed through Gabor filter network that updates 4 parameters (*c*, *σ*, *f*, and *w*) and based upon these parameters error is calculated. The minimum error corresponds to decision about the receive signal modulation.

## 4. Testing and Training of Proposed Algorithm

The training of Gabor filter network is partitioned into two phases: training of Gabor atom parameters (*c*, *σ*, *f*, and *w*) in the first phase and training the weights of adaptive filter in second phase. The parameters of Gabor atom parameters (*c*, *σ*, *f*, *w*) are tuned according to Delta rule and weights of adaptive filter are adjusted by least means square algorithm.

Let *γ*_*i*_ denote one of *i*th Gabor node parameters including shift parameter *c*_*i*_, scale parameter *σ*_*i*_, and modulation parameter *f*_*i*_. According to Delta rule,

(8) $$\begin{array}{c} \Delta \gamma_{i}=-\eta \frac{\partial J\left(k\right)}{\partial \gamma_{i}}, \end{array}$$

where *η* is learning rate.

The cost function is square of difference between desired response and output of Gabor function; that is,

(9) $$\begin{array}{c} J\left(k\right)={\left(d\left(k\right)-y\left(k\right)\right)}^{2}. \end{array}$$

The partial derivatives of cost function with respect to shift parameter *c*_*i*_, scale parameter *σ*_*i*_, and modulation para + meter *f*_*i*_ are as follows:

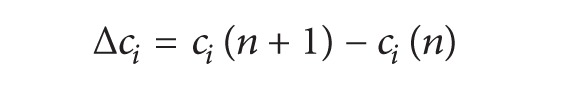
(10)

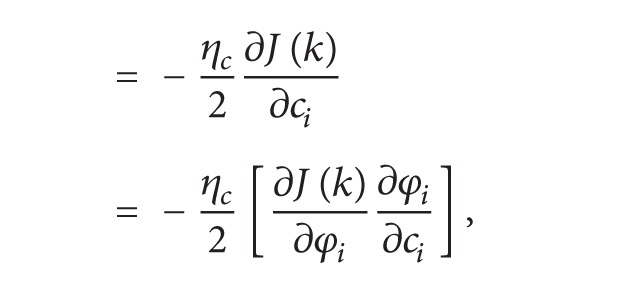
(11)

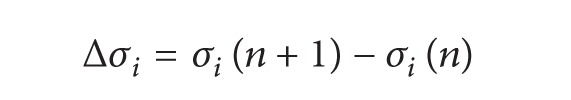
(12)

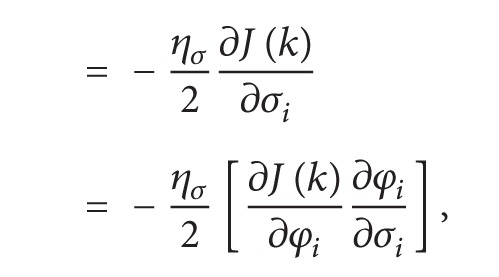
(13)

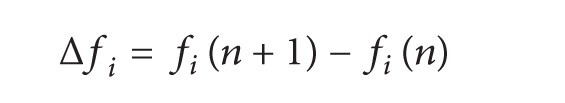
(14)

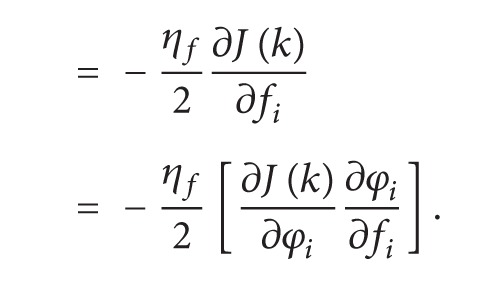
(15)
From (9),

(16) ∂J(k)∂φi=∂∂φi[(d(k)−y(k))2],∂J(k)∂φi=2[d(k)−y(k)]∂∂φi(d(k)−y(k)),∂J(k)∂φi=−2[d(k)−y(k)]∂∂φi(y(k)).

From (6),

(17) ∂∂φi(y(k))=∂∂φi[∑j=1Mφjwj],∂∂φi(y(k))=∑j=1Mδijwj,∂∂φi(y(k))=wi.

Putting (17) into (16), we get

(18) $$\begin{array}{c} \frac{\partial J\left(k\right)}{\partial \varphi_{i}}=-2{\left(d\left(k\right)-y\left(k\right)\right)w}_{i}. \end{array}$$

From (11), (13), and (15),

(19) Δci=ηc(d(k)−y(k))wi∂φi∂ci,Δσi=[ησ(d(k)−y(k))wi]∂φi∂σi,Δfi=[ηf(d(k)−y(k))wi]∂φi∂fi,gi=1σie−π((t−ci)/σi)2cos⁡⁡(fit).

From (5),

(20) $$\begin{array}{c} \varphi_{i}=\left|x_{i}\frac{1}{\sqrt{\sigma_{i}}}e^{-\pi {\left(\left(t-c_{i}\right)/\sigma_{i}\right)}^{2}}\cos\left(f_{i}t\right)\right|. \end{array}$$

For real valued signals, Gabor atom is also real, in such case.

The partial derivatives of *φ*_*i*_ with respect to shift parameter *c*_*i*_, scale parameter *σ*_*i*_, and modulation parameter *f*_*i*_ are as follows:

(21) ∂φi∂ci=∂∂ci(xigi)=∂∂ci[xi1σie−π((t−ci)/σi)2cos⁡⁡(fit)]=xiσicos⁡⁡(fit) ×[e−π((t−ci)/σi)2×−π(2(t−ciσi))(−1σi)]=xiσicos⁡⁡(fit)[e−π((t−ci)/σi)2(2π(t−ciσi2))]=xiσi5cos⁡⁡(fit)2π(t−ci)e−π((t−ci)/σi)2,∂φi∂σi=∂∂σi[xi1σie−π((t−ci)/σi)2cos⁡⁡(fit)]=xicos⁡(fit) ×[1σie−π((t−ci)/σi)2×−π(2(t−ciσi))×(−(t−ci)σi2)+e−π((t−ci)/σi)2×−12σi−3/2]=xicos⁡(fit)σie−π((t−ci)/σi)2[2π(t−ci)2σi3−12σi]∂φi∂fi=∂∂fi[xi1σie−π((t−ci)/σi)2cos⁡⁡(fit)]=−tσixie−π((t−ci)/σi)2sin⁡(fit).

The Updating of Gabor atom parameters (shift parameter *c*_*i*_, scale parameter *σ*_*i*_, and modulation parameter *f*_*i*_) according to Delta rule is as follows:

(22) Δci=ηc(d(k)−y(k))wi ×[xiσi5cos⁡⁡(fit)2π(t−ci)e−π((t−ci)/σi)2],

(23) ci(n+1)=ci(n)+ηc(d(k)−y(k))wi ×[xiσi5cos⁡⁡(fit)2π(t−ci)e−π((t−ci)/σi)2],

(24) Δσi=[ησ(d(k)−y(k))wi] ×{xicos⁡(fit)σie−π((t−ci)/σi)2×[2π(t−ci)2σi3−12σi]},

(25) σi(n+1)=σi(n)+[ησ(d(k)−y(k))wi] ×{xicos⁡(fit)σie−π((t−ci)/σi)2×[2π(t−ci)2σi3−12σi]},

(26) Δfi=[ηf(d(k)−y(k))wi] ×{−tσixie−π((t−ci)/σi)2sin⁡(fit)},

(27) fi(n+1)=fi(n)+[ηf(d(k)−y(k))wi] ×{−tσixie−π((t−ci)/σi)2sin⁡(fit)}.

Equations (23), (25), and (27) show the updated shift parameter, scale parameter, and modulation parameter of Gabor filter network.

The weights of adaptive filter are updated as follows:

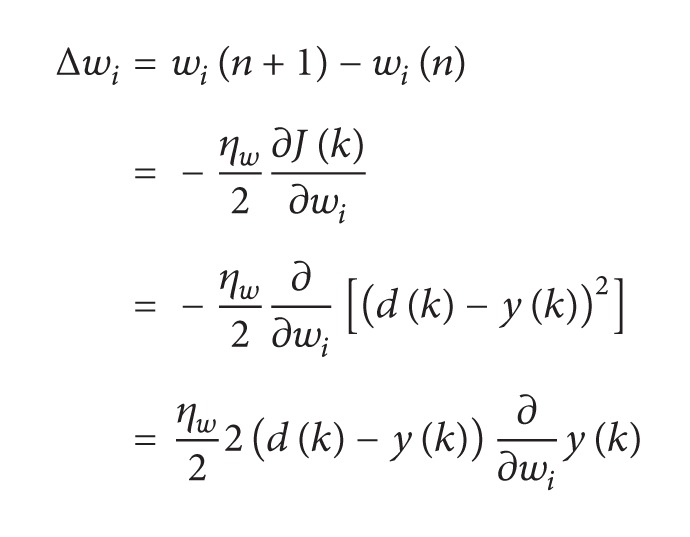
(28)

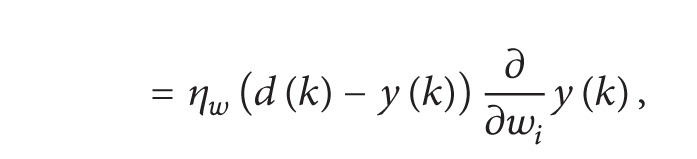
(29)

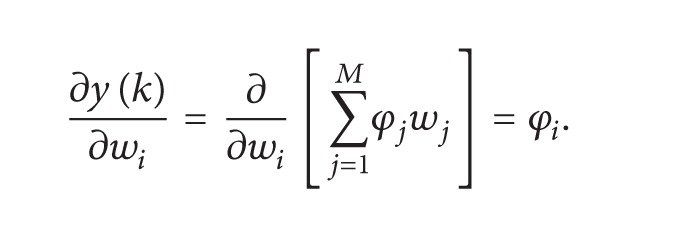
(30)
Substituting (30) in (28), we get

(31) $$\begin{array}{c} {\Delta w}_{i}=\eta_{w}\left(d\left(k\right)-y\left(k\right)\right)\varphi_{i}. \end{array}$$

From (28),

(32) $$\begin{array}{c} w_{i}\left(n+1\right)=w_{i}\left(n\right)+\eta_{w}\left(d\left(k\right)-y\left(k\right)\right)\varphi_{i}. \end{array}$$

Equation (32) shows the weight updating of the adaptive filter using least mean square algorithm.

The proposed algorithm is for feature extraction and classification of modulation formats (PSK 2 to 64, FSK 2 to 64, and QAM 2 to 64) under the influence of AWGN channel. The proposed algorithm is divided in to two phases; the first phase is for the training of Gabor filter network. In training phase, the parameters of Gabor filter network (shift, scale, and modulation) are updated according to delta rule. These parameters are now input to the adaptive filter where weights of adaptive filter are adjusted using least mean square algorithm. The error is now calculated; if error is less than the threshold, training process stops; otherwise, update the Gabor filter parameters and weights of the adaptive filter according to Delta rule and LMS algorithm until the error function is minimized. The second phase is the test phase of the algorithm, where input modulated signal is fed to the trained Gabor filter network. The parameters of Gabor filter network and weights of the adaptive filter are updated and error is calculated. The minimum error corresponds to the desired modulation format.

The proposed algorithm for training and testing of Gabor filter network for the problem of modulation classification is presented as shown in Algorithms 1 and 2.

## 5. Simulation Results

The modulation classification using Gabor filter is evaluated in this section. Firstly, the training of algorithm is presented and then the testing of algorithm in the presence of AWGN channel. The probability of correct classification (PCC) in the presence of AWGN channel is simulated here using Gabor filter network. The modulation schemes considered here are divided in three scenarios, that is, {PSK2, PSK4, PSK8, PSK16, PSK 32, and PSK64}, {FSK2, FSK4, FSK8, FSK16, FSK 32, and FSK64}, and {QAM2, QAM4, QAM8, QAM16, QAM32, and QAM64}. The PCC curves are simulated against number of iterations and SNR for three different modulation scenarios.

**Figure 4:**
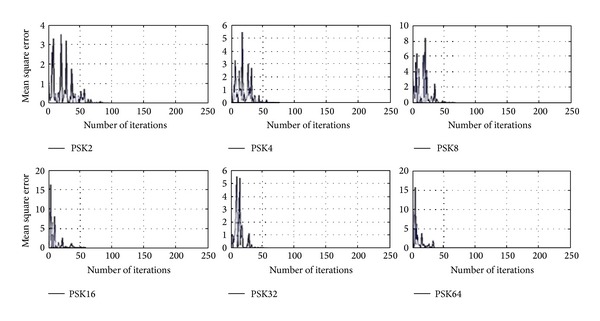
Training of Gabor filter parameters and weights for modulation classification in case of PSK modulation 2–64 for different number of iterations at SNR = 10 dB.

**Figure 5:**
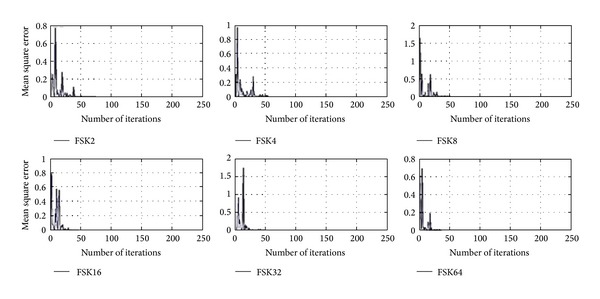
Training of Gabor filter parameters and weights for modulation classification in case of FSK modulation 2–64 for different number of iterations at SNR = 10 dB.

**Figure 6:**
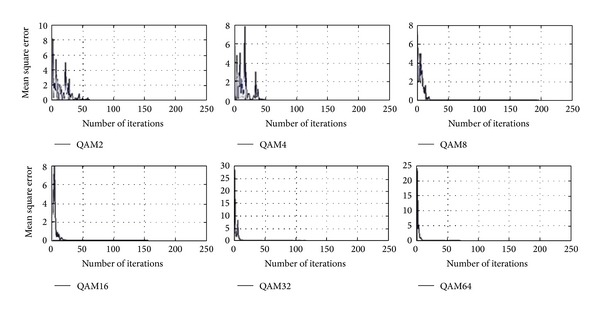
Training of Gabor filter parameters and weights for modulation classification in case of QAM 2–64 for different number of iterations at SNR = 10 dB.

**Figure 7:**
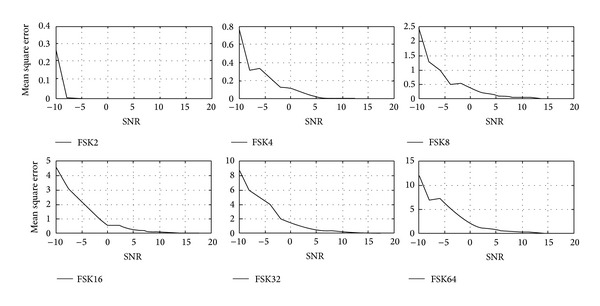
Training of Gabor filter parameters and weights for modulation classification in case of PSK modulation 2–64 at different SNRs and fixed number of iterations.

**Figure 8:**
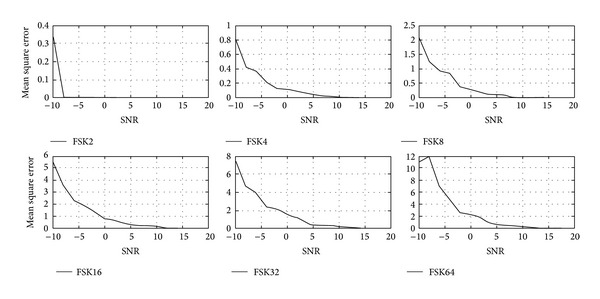
Training of Gabor filter parameters and weights for modulation classification in case of FSK modulation 2–64 at different SNRs and fixed number of iterations.

**Figure 9:**
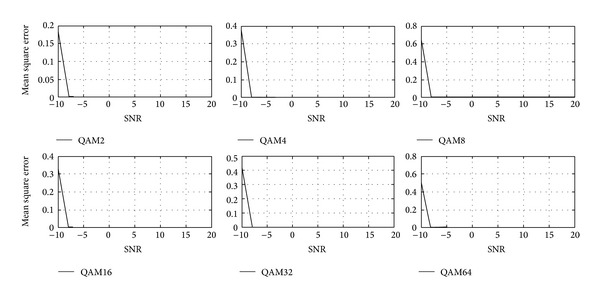
Training of Gabor filter parameters and weights for modulation classification in case of QAM 2–64 at different SNRs and fixed number of iterations.

**Figure 10:**
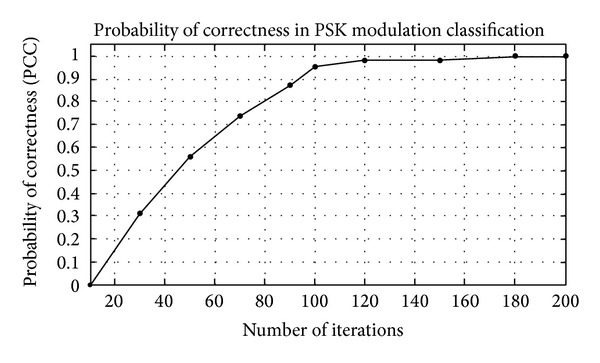
Probability of correctness (PCC) versus number of iterations at SNR = 10 dB for PSK4 example.

**Figure 11:**
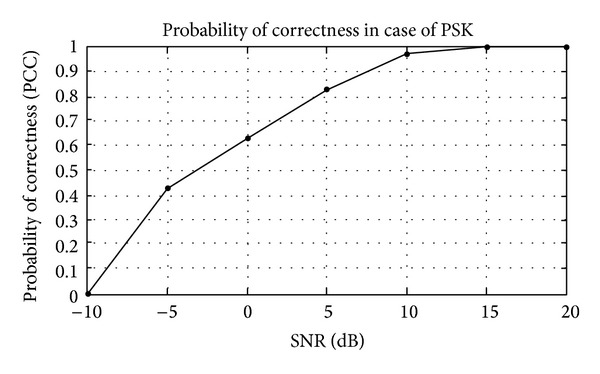
Probability of correctness (PCC) versus SNR for fixed number of iterations in PSK4 example.

**Figure 12:**
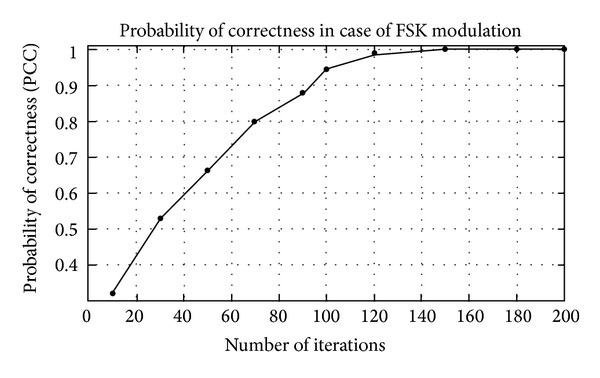
Probability of correctness (PCC) versus number of iterations at SNR = 10 dB for FSK16 example.

**Figure 13:**
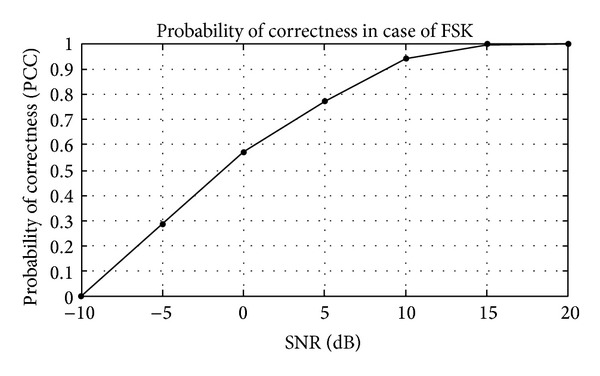
Probability of correctness (PCC) versus SNR for fixed number of iterations in FSK16 example.

**Figure 14:**
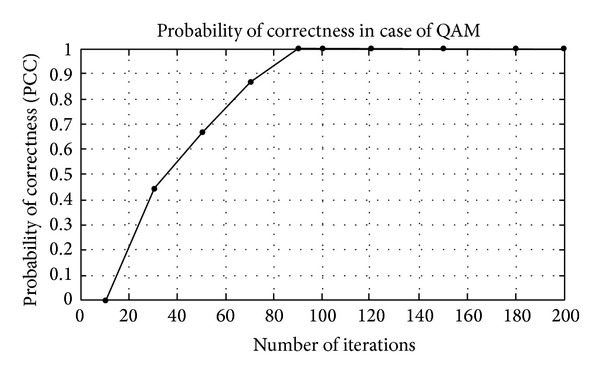
Probability of correctness (PCC) versus number of iterations at SNR = 10 dB for QAM32 example.

**Figure 15:**
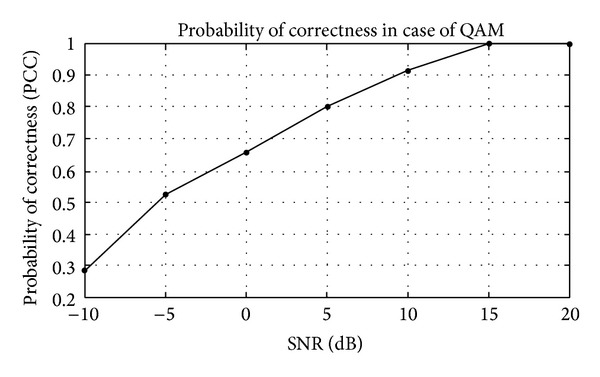
Probability of correctness (PCC) versus SNR for fixed number of iterations in QAM32 example.

Tables 1–3 and Figures 4–9 show the training of Gabor filter network for the considered modulation formats (PSK, FSK, and QAM) up to order 2 to 64. The Gabor filter network parameters (shift, scale, and modulation) are updated according to each of which considered modulation formats using delta rule and also weights are updated for each considered modulation format case using least mean square algorithm. The Gabor atom parameters and weights of Gabor filter (*c*_*i*_, *σ*_*i*_, *f*_*i*_, and *w*_*i*_) for the considered modulations are stored. The updated Gabor atom parameters and weights of Gabor filter (*c*_*i*_, *σ*_*i*_, *f*_*i*_, and *w*_*i*_) are shown in Tables 1–3. Figures 4–6 show the training of Gabor filter network for different number of iterations in case of PSK modulation, FSK modulation, and QAM, respectively. The training process for different SNRs is also shown in Figures 7–9. The training shows that the mean square error dies down as the number of iterations is increased and also by increasing the SNR. Figures 10–15 show the testing of Gabor filter network for the considered modulations formats (PSK 2 to 64, FSK 2 to 64, and QAM 2 to 64) in the presence of AWGN channel. The probability of correctness is plotted against signal to noise ratio (SNR) and different number of iterations to evaluate the classification accuracy of the proposed Gabor filter network. The simulations results show the classification accuracy for the examples of PSK4, FSK16, and QAM32 which are 100% for fixed SNR and different number of iterations.

Table 1 shows the updated Gabor atom parameters for the modulation formats of PSK of order 2 to 64. Table 1 has four parts; the first part shows the updated scale parameter for PSK 2 to 64, the second part shows the updated shift parameter, the third part shows the updated modulation parameter, and the forth part shows the updated weights of the adaptive filter. All values of updated Gabor filter parameters and weights of adaptive filter are for minimum mean square error.

Table 2 shows the updated Gabor atom parameters for the modulation formats of FSK of order 2 to 64. Table 2 has four parts; the first part shows the updated scale parameter for FSK 2 to 64, the second part shows the updated shift parameter, the third part shows the updated modulation parameter, and the forth part shows the updated weights of the adaptive filter. All values of updated Gabor filter parameters and weights of adaptive filter are for minimum mean square error.

Table 3 shows the updated Gabor atom parameters for the modulation formats of QAM of order 2 to 64. Table 3 has four parts; the first part shows the updated scale parameter for QAM 2 to 64, the second part shows the updated shift parameter, the third part shows the updated modulation parameter, and the forth part shows the updated weights of the adaptive filter. All values of updated Gabor filter parameters and weights of adaptive filter are for minimum mean square error.

Figure 4 shows the training of Gabor filter network for the case of PSK modulation format having order 2 to 64 for different number of iterations at fixed SNR of 10 dB. The parameters of Gabor filter network are trained for greater than 50 iterations for the case of PSK 2, 4, and 8, while for the PSK 16, 32, and 64 the training of Gabor filter network is for less than 50 iterations. The mean square error is minimized and approaches to zero for all curves shown in Figure 4, as a number of iterations are increased.

Figure 5 shows the Gabor filter network training for the case of FSK modulation format having order 2 to 64 for fixed number of iterations. As shown in Figure 5, the mean square error is approaching to zero, when a number of iterations are increased. The training of Gabor filter network is done for maximum 50 iterations for the FSK modulation case.

Figure 6 shows the training of Gabor filter network for QAM case with fixed SNR of 10 dB and different iterations. The training of Gabor filter network shows minimized mean square error for all curves shown in Figure 6 with less number of iterations. In Figure 6, QAM 16, 32, and 64 are trained for 20 iterations and QAM 2, 4, and 8 are trained for above 50 iterations.

Figure 7 shows the training of Gabor filter network parameters and weights of adaptive filter in case of PSK modulation up to order 2 to 64 with fixed number of iterations and different SNRs. As signal to noise ratio is varied from 0 to 20, the mean square error approaching towards zero. The training of proposed algorithm for all cases of considered modulation is done successfully and Figure 7 shows that the proposed algorithm for the modulation classification is trained at SNR of 10 dB.

Figure 8 shows the training of Gabor filter network parameters and weights of adaptive filter in case of FSK modulation up to order 2 to 64 with fixed number of iterations and different SNRs. The training of FSK modulation formats is done at SNR of 10–15 dB.

The training of Gabor filter network for QAM 2 to 64 at different SNRs and fixed number of iterations is shown in Figure 9. The parameters of Gabor filter network and weights of the adaptive filter are updated and mean square error is minimized as SNR is increased from 0 to 20 dB.

The example considered in Figures 10 and 11 is PSK4. The probability of correctness versus different number of iterations at SNR = 10 dB is shown in Figure 10, while PCC curve versus SNR at fixed number of iterations is shown in Figure 11. The PCC in Figure 10 is approximately 1 when the number of iterations is increased up to 200. From Figures 10 and 11, the classification performance of Gabor filter network for the PSK modulation scenario under the effect of white Gaussian noise channel is approximately 100% at lower SNR. In Figures 10 and 11, the example considered are PSK4 and it is shown form the results that PSK4 classified correctly among class of PSK modulation formats having order 2 to 64.

In Figures 12 and 13, the probability of correctness for FSK modulation scenario is shown for different number of iterations and SNR. The example considered is FSK16. The PCC curve shows that the classification performance is approximately 100% at SNR = 15 dB for fixed number of iterations. The probability of correctness is approximately 1 in Figure 12, when a number of iterations are increased up to 200. The FSK 16 example is classified accurately among the group of considered modulation formats.

In Figures 14 and 15, the example considered is QAM32 for classification purpose. The classification curve in the form of PCC versus different number of iterations at fixed SNR of 10 dB is shown in Figure 14, while in Figure 15 PCC curves versus SNR at fixed number of iterations are shown. The classification of Gabor filter network in case of QAM is quite better with the existing modulation classification techniques.

The simulation results show the 100% classification accuracy of the proposed algorithm. The features extracted from the proposed architecture and classifier based upon Gabor filter network provide correct classification among group of considered modulation formats. Moreover, the received signal is corrupted by additive white Gaussian noise but the classification accuracy is approximately 100% at lower SNRs. The algorithm is also computationally less complex and classification accuracy is attained at less number of iterations.

## 6. Conclusion

In this paper, proposed classifier which is Gabor filter based is used for feature extraction and also for the classification purpose. The Gabor filter input layer constitutes the feature extraction part (*c*_*i*_, *σ*_*i*_, and *f*_*i*_), whereas weights (*w*_*i*_) between Gabor atom nodes and output of the Gabor filter constitute the linear classification part. The feature extraction layer and classification part search for the optimal Gabor atom parameters and weights of the Gabor filter, so that error is to be minimized. The considered modulations such as PSK2, PSK4, PSK8, PSK16, PSK32, PSK64, QAM2, QAM4, QAM8, QAM 16, QAM 32, QAM 64, FSK2, FSK4, FSK8, FSK16, FSK32, and FSK64 are classified under the effects of AWGN channel. The classifier proposed here is very effective performance in considered scenarios of modulation. The proposed novel Gabor filter based modulation classification technique shows 100% classification accuracy at lower SNR.

## Figures and Tables

**Algorithm 1:**
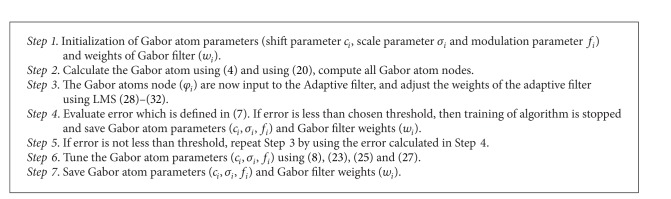
(Training of Gabor filter Network for Modulation Classification).

**Algorithm 2:**
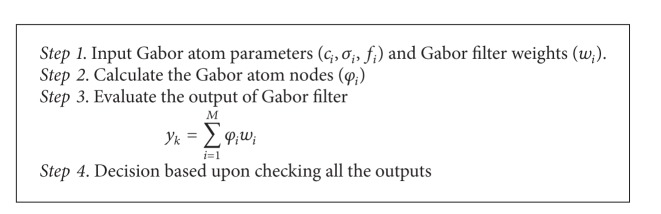
(Testing of Gabor filter Network for Modulation Classification).

**Table 1:** Updated Gabor filter atom parameters and weights for PSK modulation 2–64.

Shift parameter (*c*)					
PSK2	PSK4	PSK8	PSK16	PSK32	PSK64
5.54	5.07	5.77	5.65	4.81	5.27
5.77	5.90	4.25	4.45	4.18	4.29
4.69	4.24	4.33	5.20	4.56	5.56
4.63	4.72	4.83	4.17	4.67	4.56
5.02	4.29	5.00	4.08	5.13	4.59
5.03	5.18	5.68	5.89	5.23	4.79
5.16	5.09	5.89	4.91	5.79	4.63
4.61	4.67	5.52	5.91	4.35	4.47
4.48	4.59	5.13	5.62	5.61	4.47
5.43	5.14	5.13	4.94	4.15	4.75

Scale parameter (σ)					
PSK2	PSK4	PSK8	PSK16	PSK32	PSK64

15.63	16.25	10.25	16.76	1.67	2.45
16.03	8.57	8.17	5.18	13.53	16.21
14.87	14.08	2.55	18.87	6.78	5.33
1.92	18.27	12.31	6.63	13.33	4.66
6.44	3.16	11.89	18.48	9.38	5.94
13.32	3.17	4.80	6.85	11.90	2.50
11.49	12.94	12.83	15.33	7.44	15.29
14.39	3.33	7.33	7.58	6.03	5.21
19.04	14.23	7.39	5.13	18.00	12.81
8.67	2.94	8.48	1.05	12.56	2.59

Modulation parameter (*f*)					
PSK2	PSK4	PSK8	PSK16	PSK32	PSK64

2.35	−0.05	0.80	3.14	−0.53	1.82
−2.72	−0.56	−0.37	−0.53	2.90	−3.02
1.91	2.52	−1.90	0.95	−1.33	−2.24
−1.02	0.10	−1.55	1.46	2.47	0.38
1.73	1.08	1.83	1.51	−0.75	−1.89
−2.75	−1.83	−2.57	−1.42	2.66	1.10
0.47	0.20	−2.79	−2.21	0.54	−1.57
0.32	0.11	−0.93	−1.56	−0.94	−0.55
1.88	0.33	−2.59	0.31	−1.28	0.68
−0.76	−2.27	0.07	2.83	−1.01	−1.70

Weights (*w*)					
PSK2	PSK4	PSK8	PSK16	PSK32	PSK64

5.059	−2.625	−0.747	−0.119	0.837	−4.310
−1.823	−1.927	0.671	0.297	1.309	−3.073
6.844	−2.482	−0.533	−0.371	−3.033	8.333
1.847	2.528	0.499	−0.356	−0.894	−3.802
5.384	−0.090	0.677	−0.346	−1.484	1.413
8.671	1.923	0.256	−0.917	−0.843	3.887
0.058	−2.857	−0.703	−0.455	0.449	−1.762
−1.356	1.852	−0.555	−0.487	−1.844	2.682
1.899	1.055	0.481	−1.498	0.844	−3.901
−3.603	−2.714	0.402	−0.664	1.832	2.549

**Table 2:** Updated Gabor filter atom parameters and weights for FSK modulation 2–64.

Shift parameter (*c*)					
FSK2	FSK4	FSK8	FSK16	FSK32	FSK64
4.25	4.36	5.35	4.67	5.74	4.22
4.09	4.88	5.80	4.20	4.55	5.93
4.58	4.59	5.54	5.94	4.01	5.89
5.50	5.81	5.71	5.35	5.57	5.25
4.46	5.55	4.60	5.83	4.59	5.96
5.36	4.93	4.28	4.01	5.50	5.89
4.27	4.49	4.16	4.31	5.15	5.95
4.38	5.28	4.75	5.70	5.76	5.40
4.35	5.03	4.41	5.04	5.39	4.77
4.38	4.29	5.58	5.97	5.31	5.84

Scale parameter (σ)					
FSK2	FSK4	FSK8	FSK16	FSK32	FSK64

4.44	6.46	6.80	17.24	19.65	12.27
14.22	2.53	3.57	3.73	6.25	19.22
2.11	8.02	15.44	10.10	17.67	6.86
5.89	10.94	4.75	11.42	4.03	6.93
19.21	2.30	11.89	2.10	6.77	19.96
19.44	2.78	14.26	5.46	4.12	14.04
17.74	4.84	11.82	10.50	3.96	5.87
1.37	6.67	10.98	17.24	17.69	5.87
19.53	15.99	13.96	8.73	19.91	3.74
12.67	3.57	1.45	14.57	3.65	19.59

Modulation parameter (*f*)					
FSK2	FSK4	FSK8	FSK16	FSK32	FSK64

−0.15	−2.97	2.87	1.64	2.42	−1.70
1.15	2.77	0.84	0.76	−1.48	−2.07
1.22	1.82	2.69	−2.54	−1.23	−0.84
−1.50	2.91	−0.67	2.37	0.77	3.10
2.87	1.94	−0.99	−2.95	0.16	0.45
0.66	2.08	2.29	0.04	−3.00	−2.56
−2.45	−0.72	2.18	−1.43	−1.85	0.41
0.19	2.87	−1.23	2.59	2.05	−1.09
−0.44	1.69	−1.04	−2.30	0.69	0.34
2.55	−0.76	1.41	−1.80	0.79	−0.54

Weights (*w*)					
FSK2	FSK4	FSK8	FSK16	FSK32	FSK64

−2.579	−7.096	0.931	0.124	−0.318	8.540
−0.490	−0.572	−1.195	−0.239	−1.668	5.174
−1.532	−3.564	−1.195	3.627	4.663	−1.570
6.719	−5.807	−1.110	−0.984	−2.764	0.566
−9.317	4.059	−0.083	−0.283	0.840	−2.799
−8.051	−2.456	0.068	0.990	3.381	−1.628
0.012	3.768	0.443	0.242	4.211	12.898
−12.209	1.989	−1.487	1.298	−1.675	−6.270
8.351	5.954	−1.451	−0.296	−4.969	4.876
−4.070	−0.026	0.402	1.027	−5.423	−8.421

**Table 3:** Updated Gabor filter atom parameters and weights for QAM 2–64.

Shift parameter (*c*)					
QAM2	QAM4	QAM8	QAM16	QAM32	QAM64
5.54	5.07	5.77	5.65	4.81	5.27
5.77	5.90	4.25	4.45	4.18	4.29
4.69	4.24	4.33	5.20	4.56	5.56
4.63	4.72	4.83	4.17	4.67	4.56
5.02	4.29	5.00	4.08	5.13	4.59
5.03	5.18	5.68	5.89	5.23	4.79
5.16	5.09	5.89	4.91	5.79	4.63
4.61	4.67	5.52	5.91	4.35	4.47
4.48	4.59	5.13	5.62	5.61	4.47
5.43	5.14	5.13	4.94	4.15	4.75

Scale parameter (σ)					
QAM2	QAM4	QAM8	QAM16	QAM32	QAM64

15.63	16.25	10.25	16.76	1.67	2.45
16.03	8.57	8.17	5.18	13.53	16.21
14.87	14.08	2.55	18.87	6.78	5.33
1.92	18.27	12.31	6.63	13.33	4.66
6.44	3.16	11.89	18.48	9.38	5.94
13.32	3.17	4.80	6.85	11.90	2.50
11.49	12.94	12.83	15.33	7.44	15.29
14.39	3.33	7.33	7.58	6.03	5.21
19.04	14.23	7.39	5.13	18.00	12.81
8.67	2.94	8.48	1.05	12.56	2.59

Modulation parameter (*f*)					
QAM2	QAM4	QAM8	QAM16	QAM32	QAM64

2.35	−0.05	0.80	3.14	−0.53	1.82
−2.72	−0.56	−0.37	−0.53	2.90	−3.02
1.91	2.52	−1.90	0.95	−1.33	−2.24
−1.02	0.10	−1.55	1.46	2.47	0.38
1.73	1.08	1.83	1.51	−0.75	−1.89
−2.75	−1.83	−2.57	−1.42	2.66	1.10
0.47	0.20	−2.79	−2.21	0.54	−1.57
0.32	0.11	−0.93	−1.56	−0.94	−0.55
1.88	0.33	−2.59	0.31	−1.28	0.68
−0.76	−2.27	0.07	2.83	−1.01	−1.70

Weights (*w*)					
QAM2	QAM4	QAM8	QAM16	QAM32	QAM64

−0.031	−0.504	1.009	−1.428	−1.095	0.339
−0.021	−0.446	−0.796	−1.339	−0.559	−0.835
−0.043	−0.187	−0.285	−0.725	0.304	0.442
0.194	0.794	0.299	−1.122	1.359	−0.558
0.354	1.061	0.664	−0.476	−1.109	0.506
0.250	−0.591	−0.306	0.044	−1.357	−0.234
0.097	−0.622	−0.380	1.193	0.290	0.022
0.007	0.247	−0.769	−0.316	0.624	−0.177
0.245	0.467	−0.608	0.984	−0.334	0.694
0.046	−0.117	0.536	0.552	−0.294	0.423
